# An Efficient Frequency Recognition Method Based on Likelihood Ratio Test for SSVEP-Based BCI

**DOI:** 10.1155/2014/908719

**Published:** 2014-08-28

**Authors:** Yangsong Zhang, Li Dong, Rui Zhang, Dezhong Yao, Yu Zhang, Peng Xu

**Affiliations:** ^1^School of Computer Science and Technology, Southwest University of Science and Technology, Mianyang 621010, China; ^2^Key Laboratory for NeuroInformation of Ministry of Education, School of Life Science and Technology, University of Electronic Science and Technology of China, Chengdu 610054, China; ^3^Key Laboratory for Advanced Control and Optimization for Chemical Processes, Ministry of Education, East China University of Science and Technology, Shanghai 200237, China

## Abstract

An efficient frequency recognition method is very important for SSVEP-based BCI systems to improve the information transfer rate (ITR). To address this aspect, for the first time, likelihood ratio test (LRT) was utilized to propose a novel multichannel frequency recognition method for SSVEP data. The essence of this new method is to calculate the association between multichannel EEG signals and the reference signals which were constructed according to the stimulus frequency with LRT. For the simulation and real SSVEP data, the proposed method yielded higher recognition accuracy with shorter time window length and was more robust against noise in comparison with the popular canonical correlation analysis- (CCA-) based method and the least absolute shrinkage and selection operator- (LASSO-) based method. The recognition accuracy and information transfer rate (ITR) obtained by the proposed method was higher than those of the CCA-based method and LASSO-based method. The superior results indicate that the LRT method is a promising candidate for reliable frequency recognition in future SSVEP-BCI.

## 1. Introduction

Brain-computer interface (BCI) can provide online communication between a human or animal brain and external devices without depending on the normal output pathways of peripheral nerves and muscles [1]. Research interest has increased because of its potential application value in neural engineering and neuroscience [2, 3]. Many EEG signals could serve as control signals in BCI systems [1, 3]. In recent years, steady-state visual evoked potentials (SSVEPs) have been widely used in SSVEP-based BCI [2, 4–6]. SSVEP has the same fundamental frequency as well as harmonics of the flickering visual stimulus, and it has high signal-to-noise ratio (SNR) and stable spectrum [7]. Accordingly, SSVEP-based BCI usually requires little training effort and achieving high information transfer rate (ITR) and becomes an important branch for designing BCI applications [8, 9].

In the SSVEP-based BCI system, the targets are encoded by a single frequency or various combinations of frequencies. A command can be transmitted by shifting the subject's attention to the corresponding target. Although SSVEP has the aforementioned characteristics, it is likely to be contaminated by spontaneous EEG activities and other noises [10, 11]. How to efficiently recognize the target with short time window and low error rate is one of the key topics to boost the IRT of the SSVEP-based BCI systems. Therefore, it is necessary to develop an efficient frequency recognition method for SSVEP-based BCI. The existing traditional recognition methods are power spectral density analysis (PSDA) [12] and stability coefficients (SC) [10], which are mainly based on the single EEG channel. These methods are sensitive to noise and need long time window to perform the recognition, which may limit the real-time performance of SSVEP-based BCIs. In addition, because users usually have shown large intervariation in the SSVEP amplitude and distribution, additional calibration is required for parameter optimization (e.g., channel selection and appropriate data length) with these traditional methods [8, 11].

To overcome the drawbacks in the single channel based recognition, the multichannel recognition methods have aroused wide interests. Lin et al. proposed a method based on the canonical correlation analysis (CCA) [13]. Another multichannel recognition method is minimum energy combination (MEC) method proposed by Friman and his colleagues [11]. These two methods showed superior performance as compared to the traditional recognition methods [8, 9]. In addition, Nan and his colleagues had shown that CCA-based method could achieve better performance than MEC [14], and CCA has been widely adopted for frequency recognition in SSVEP-based BCI systems. Zhang et al. using LASSO-based frequency recognition method showed that the sparse regression model greatly improved the classification performance over CCA [15]. Therefore, in current work, we will use the CCA-based method and the LASSO-based method as the baselines to evaluate the performance of the proposed method by us.

LRT is a tool to test the independence of two sets of multivariate variables [16]. In this paper, we proposed a novel frequency recognition method based on likelihood ratio test (LRT) to further improve the frequency recognition accuracy for SSVEP-BCIs. For the first time, the LRT was utilized to calculate the correlation between the multichannel EEG signals and the reference signals. Experimental results based on the simulation and the real EEG data from eleven subjects demonstrate that the proposed method showed better performance as compared to the CCA-based method and the LASSO-based method.

## 2. Materials and Methods

This study was approved by the Institution Research Ethics Board at the University of Electronic Science and Technology of China. All participants were asked to read and sign an informed consent form before participating in the study. All the participants received monetary compensation for their time and effort following completion of the experiment.

### 2.1. LRT-Based Frequency Recognition

Suppose that $X=\left[\begin{array}{c} X_{1}\\ X_{2} \end{array}\right]$ is a *p*-dimensional normal distribution vector. *X*_1_ and *X*_2_ are *p*_1_- and *p*_2_-dimensional vector, respectively, and *p* = *p*_1_ + *p*_2_. The vector mean *μ* and covariance matrix ∑ of *X* are given by

(1) $$\begin{array}{c} \mu =\left[\begin{array}{c} \mu_{1}\\ \mu_{2} \end{array}\right],\;\;\sum =\left[\begin{array}{cc} \sum_{11} & \sum_{12}\\ \sum_{21} & \sum_{22} \end{array}\right]. \end{array}$$

The null hypothesis of two independent sets of variables *X*_1_ and *X*_2_ is represented as

(2) $$\begin{array}{c} H_{0}:\sum_{12}=0,\;\;H_{1}:\sum_{12}\neq 0. \end{array}$$

Suppose that *x*_1_, *x*_2_,…, *x*_*N*_ are the samples drawn from *X*. According to likelihood ratio test [16], the likelihood ratio statistic for formula (2) is as follows:

(3) $$\begin{array}{c} \lambda =\frac{\text{ma}{\text{x}}_{H_{0}}L\left(\mu ,\sum \right)}{\text{ma}{\text{x}}_{H_{0}\cup H1}L\left(\mu ,\sum \right)}=\frac{{\left|\hat{\sum }\right|}^{(1/2)N}}{{\left|{\hat{\sum }}_{11}\right|}^{(1/2)N}\cdot {\left|{\hat{\sum }}_{22}\right|}^{(1/2)N}}, \end{array}$$

where

(4) ∑^=1N∑k=1N(xk−x−)(xk−x−)T,  x−=1N∑k=1Nxk,∑ij^=1N∑k=1N(xki−x−i)(xkj−x−j)T,x−i=1N∑k=1Nxki, i,j=1,2.

*T* denotes the complex conjugate transpose of vectors or matrices. Then we have the statistical measurement *λ* = *V* ^*N*/2^, where

(5) $$\begin{array}{c} V=\frac{\left|\hat{\sum }\right|}{\left|{\hat{\sum }}_{11}\right|\cdot \left|{\hat{\sum }}_{22}\right|}=\frac{\left|{\hat{\sum }}_{11}-{\hat{\sum }}_{12}\cdot {\hat{\sum }}_{22}^{-1}\cdot {\hat{\sum }}_{21}\right|}{\left|{\hat{\sum }}_{11}\right|}. \end{array}$$

In fact, *V* is also a metric to measure the coefficient of alienation between *X*_1_ and *X*_2_ [16]. Therefore, a measure of association is 1 minus *V*. Furthermore, the association can be modified to take account of the number of dimensions [16]. Accordingly, we can get the measure of association as follows:

(6) $$\begin{array}{c} C=1-{\left(\frac{\left|\hat{\sum }\right|}{\left|{\hat{\sum }}_{11}\right|\cdot \left|{\hat{\sum }}_{22}\right|}\right)}^{1/p_{2}}. \end{array}$$

*C* ranges from 0 to 1. If *X*_1_ and *X*_2_ are identical, then *C* = 1. If *X*_1_ and *X*_2_ are independent, *C* = 0.

Suppose that *X* is the multichannel SSVEP data and *Y*_1_, *Y*_2_,…, *Y*_*M*_ are the reference signal matrices which are formed by the following formula at the stimulus frequencies *f*_1_, *f*_2_,…, *f*_*M*_:

(7) Ym=(sin(2π·fm·t)cos⁡(2π·fm·t)⋮sin(2π·Nh·fm·t)cos⁡(2π·Nh·fm·t)),t=1Fs,2Fs,…,NFs, m=1,…M,

where *N*_*h*_ is the number of harmonics and *F*_*s*_ is the sampling rate.

For frequency recognition, we can calculate the correlation coefficients (*C*_1_, *C*_2_,…, *C*_*M*_) between *X* and *Y*_1_, *Y*_2_,…, *Y*_*M*_ with the formulas (4)–(6). Then the frequency of *X* is recognized as the stimulus frequency corresponding to the maximal correlation coefficient.

### 2.2. CCA-Based Frequency Recognition

CCA is a multivariable statistical method to explore the underlying correlation between two sets of variables [17]. When using CCA for frequency recognition, we also require the reference signals described in formula (7) [13]. With CCA, we can find the weight vectors *W*_*x*_ and *W*_*y*_ to obtain the maximum canonical correlation between *x* = *X* ^*T*^ *W*_*x*_ and *y* = *Y*_*m*_^*T*^ *W*_*y*_ (*m* = 1,2,…, *M*) by solving the following optimization problem:

(8) $$\begin{array}{c} \underset{W_{x},W_{r}}{\max}\rho \left(x,y\right)=\frac{W_{x}^{T}X^{T}Y_{m}W_{y}}{\sqrt{W_{x}^{T}\cdot XX^{T}\cdot W_{x}^{T}\cdot W_{y}^{T}\cdot Y_{m}Y_{m}^{T}\cdot W_{y}}}. \end{array}$$

For *X* and each reference signal *Y*_*m*_ (*m* = 1,2,…, *M*), we can obtain a maximum canonical correlation *ρ*_*m*_ by formula (8) and use these coefficients to recognize the frequency of *X*. Similar to LRT-based method, the frequency of *X* is the stimulus frequency corresponding to the maximal correlation coefficient [13].

### 2.3. LASSO-Based Frequency Recognition

LASSO-based stimulus frequency recognition model showed that the sparse regression model greatly improved the classification performance over CCA [15]. For a SSVEP response *x*, and the design matrix *Y* = [*Y*_1_ *Y*_2_ … *Y*_*M*_], we can model a linear regression model by adding the noise as follows:

(9) $$\begin{array}{c} x=Y\beta +\varepsilon , \end{array}$$

where *Y*_1_, *Y*_2_,…, *Y*_*M*_ are the reference signal matrices corresponding to *M* stimulus frequencies, respectively, *β* represents the regression coefficients, and *ε* represents a noise vector with zero mean and unit variance. For the LASSO estimate, the *β* is given by the following formula:

(10) $$\begin{array}{c} \hat{\beta }=\arg\underset{\beta }{\min}\left({||x-Y\beta ||}_{2}^{2}+\lambda {||\beta ||}_{1}\right), \end{array}$$

where ||||_2_ and ||||_1_ denote the *l*_2_-norm and *l*_1_-norm, respectively. *λ* is a penalty parameter which controls the sparsity of solution $\hat{\beta }$. The function “lasso.m” in Matlab (MathWorks) was used as implement to calculate the $\hat{\beta }$.

Here, the number of harmonics was two, and the number of the stimulus frequency was 4. Therefore, we can denote $\hat{\beta }$ as $\hat{\beta }=[\beta_{1,1},\beta_{1,2},\beta_{1,3},\beta_{1,4},\ldots ,\beta_{4,1},\beta_{4,2},\beta_{4,3},\beta_{4,4}]$. For each channel, we could get a $\hat{\beta }$ (*i* = 1,…*Nc*). After that, the contribution degree (CD) of *i*th stimulus frequency and its harmonic to the EEG signal can be calculated as follows:

(11) $$\begin{array}{c} CD_{i}=\frac{\sum_{k=1}^{N_{c}}\sum_{j=1}^{2N_{h}}\left|\beta_{i,j}^{k}\right|}{N_{c}}, \end{array}$$

where *N*_*c*_ is the number of the used channels and *N*_*h*_ is the number of harmonics.

Similar to CCA-based method, the frequency of *X* is the stimulus frequency corresponding to the maximal contribution degree [15]. For the details about the LASSO-based method, please refer to the reference [15].

### 2.4. Simulation

The main purpose of this simulation was to study the antinoise capability of CCA-based method, LASSO-based method, and LRT-based method. We chose four frequencies, that is, 7.5 Hz, 8.6 Hz, 10 Hz, and 12 Hz, to simulate the SSVEP signals. For each frequency, we generated 8 sinusoidal signals to simulate 8 channels of SSVEPs. The sampling rate was 250 Hz, and the signals lasted for 10 seconds. Then, Gaussian white noise was added to the sinusoidal signals to simulate the noise-contaminated signals. Finally, the two methods were used for frequency recognition, and the time window length was 1 s as that used in the simulation by Lin et al. [13]. The accuracy was used to evaluate the recognition result, which was the ratio of the number of correct recognition operations to the 40 recognition operations. At each SNR level, the procedure was repeated 50 times, and the mean accuracies across 50 runs are reported. To show the influence of the SNR on the accuracy of these methods, SNRs ranging from −7 db to −20 db were considered to add the noises. The SNR is defined as follows:

(12) $$\begin{array}{c} \text{SNR}=10\log\frac{P_{\text{signal}}}{P_{\text{noise}}}=10\log\frac{\left(A/\sqrt{2}\right)}{\sigma^{2}}, \end{array}$$

where *P*_signal_ and *P*_noise_ are the power of the signal and the power of the noise, respectively. *A* is the amplitude of the sinusoidal signals, and *σ* ^2^ is the variance of the noise [13, 14].

### 2.5. Offline Experiment

To further evaluate the performance of the three methods, the real SSVEP data was also used. The SSVEP data was from an offline SSVEP-based BCI experiment in our lab with 4 frequencies, that is, 7.5 Hz, 8.6 Hz, 10 Hz, and 12 Hz. The flickering stimulus was presented by a computer through a control program realized by C++Builder and Windows DirectX API. A laptop with a 13′′ screen and a 60 Hz refresh rate was used to present the stimuli.

The experiment was performed in a normal room. EEG signals were recorded from the scalp via 8 Ag/AgCl recording electrodes with a Symtop Amplifier (NIL System, Chengdu, China). The electrodes were placed at P3, P4, O1, O2, Pz, Oz, PO7, and PO8. Fcz and Afz were adopted as the reference and ground, respectively. Data were sampled at 1000 Hz and filtered with a band-pass filter from 0.5 to 30 Hz and a 50 Hz notch filter. Impedances were kept below 5 kΩ. Eleven healthy right-handed subjects (two female and nine male, age ranging from 21 to 25 years) participated in this study. All subjects had normal or corrected-to-normal vision. These subjects did not have any history of epileptic seizure or mental disease. Six were naive to the SSVEP-based BCI equipment and paradigm. During the experiment, the subjects were seated in a comfortable armchair, 60 cm away from the center of the laptop monitor. The subjects were instructed to gaze binocularly at each frequency flickering stimulus for 30 s, followed by a rest period of approximately 1-2 min.

Based on the EEG data, we evaluated the performances of the three methods using different time window, that is, 0.5 s, 0.75 s, 1 s, 1.25 s, 1.5 s, 1.75 s, and 2 s. For each time window, we extracted nonoverlapping segments from the 30 s data of each frequency and pooled all the segments for the four frequencies together. Afterward, we used the three methods to conduct the frequency recognition. The accuracy, which was the ratio of the number of segments correctly classified to the number of total segments, was used to evaluate the performances of the three methods.

### 2.6. Information Transfer Rate (ITR)

In this study, information transfer rate (ITR) was adopted as the criteria to evaluate the BCI system [1]. If *N* possible selections exist in one trial, if each selection is of the identical probability to be selected by the user, if the probability (*P*) that the desired selection will actually be selected is always the same, and each of the other (i.e., undesired) selections has the same probability of being selected (i.e., (1 − *P*)/(*N* − 1)), then the bit rate (in bits min^−1^) can be computed as follows:

(13) $$\begin{array}{c} \text{Bt}=\text{lo}{\text{g}}_{2}N+P\text{lo}{\text{g}}_{2}P+\left(1-P\right)\times \text{lo}{\text{g}}_{2}\left[\frac{1-P}{N-1}\right]. \end{array}$$

Then, the ITR (bits/min) is equal to Bt multiplied by the selection speed (i.e., trials per minute).

For our offline analysis, we used a simulation method to conduct a simulated online ITR test [4]. A 0.5 s was set to simulate the interval which was given to the subjects to shift gaze as the online situation. Therefore, a trial period was 0.5 plus the window length which was used to obtain a frequency recognition result.

## 3. Results

**Figure 1:**
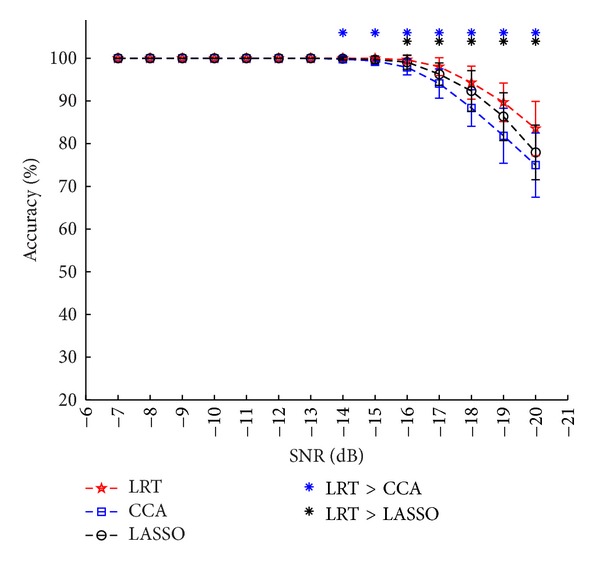
Simulation recognition accuracies and standard deviation of the three methods at different SNR levels. The asterisk denotes a significant difference between two methods (paired *t*-test, *P* < 0.05). The error bars represent standard deviations. A time window length of 1 s was used for recognition.

For the simulation, Figure 1 shows the average recognition accuracies of the 2 methods at various SNR levels. The LRT-based method significantly differed from the CCA-based method when the SNR was lower than −13 db and from the LASSO-based methods when the SNR was lower than −15 db, which demonstrates that the LRT-based method showed higher accuracy and better robustness to decreased SNRs.

**Figure 2:**
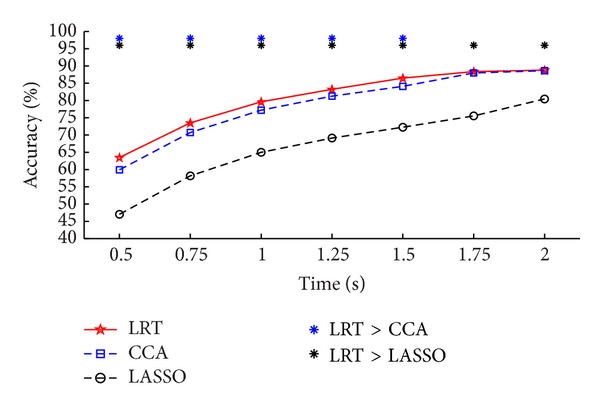
Average accuracies of the three methods at different time windows. The asterisk denotes the significant difference between two methods (paired *t*-test, *P* < 0.05).

**Figure 3:**
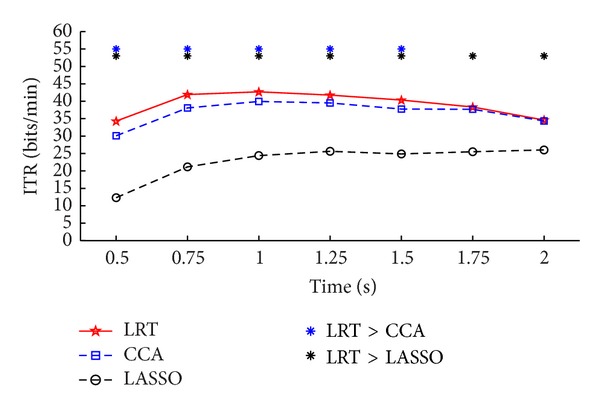
Average ITR of the three methods at different time windows. The asterisk denotes the significant difference between two methods (paired *t*-test, *P* < 0.05).

For the offline EEG data, Table 1 summarizes the recognition accuracies and Table 2 shows the ITR for the eleven subjects with different time window lengths by the three methods. At each time window length, most of the subjects showed better performance by LRT-based method than the other two methods. Figures 2 and 3 present the paired *t*-test significance test results for the recognition accuracies and ITR of the three methods, respectively. The results show that the proposed method is significantly better than the CCA-based method at most time window lengths, especially for the shorter time window lengths. It also suggests that our method is more efficient and robust than CCA-based method. From Figures 2 and 3, the results of the LASSO-based method were worse than those of the CCA-based method, which was different from the simulation result as in Figure 1 (not consistent with the results in [15]). The use of signals within a broader area can introduce more noise and negatively impact recognition accuracy. Multichannel detection methods, that is, the LRT-based and the CCA-based methods, benefit from an optimized combination of multiple signals and have greater robustness against noise, thus improving the results. For the LASSO-based method, it calculates the classification features from each channel independently, such that those features from the low SNR channels can deteriorate its recognition performance.

## 4. Discussion and Conclusion

Efficient frequency recognition is critical for a high performance SSVEP-based BCI system. The popular multichannel frequency recognition methods benefit from an optimized combination of multiple signals and have better robustness against noise. These methods always achieve higher recognition accuracy and increase the convenience of the BCI system for users due to the nonrequirement for specific channel selection and data calibration [8, 9, 11, 13].

In this study, a multichannel frequency recognition method based on LRT is proposed, which adopts LRT to calculate the correlation coefficient between EEG data and reference signals for frequency recognition. From the simulation and offline experiment, we could see that LRT-based method can achieve higher recognition accuracy in shorter time window and is of better robustness against noise than the CCA-based method and the LASSO-based method. The accurate detection of the intention of the user with short data lengths is crucial for developing a high-performance SSVEP-based BCI system [10]. Furthermore, short data acquisition can prevent fatigue to some extent because of shorter gazing time. In current study, we just demonstrated the superiority of LRT based on offline analysis. In the future, we will realize LRT-based method in the online BCI system to further test its online performance.

**Figure 4:**
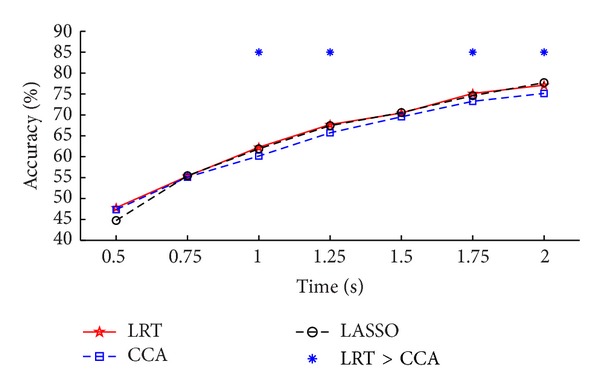
Average accuracies of the three methods at different time window lengths with the three channels (O1, Oz, and O2). The asterisk denotes the significant difference between two methods (paired *t*-test, *P* < 0.05).

For the LASSO-based method, it can yield better performance in extracting robust and detectable features of SSVEP, and the ITR obtained by the LASSO model is significantly higher than that of the CCA-based method when only three channels O1, O2, and Oz are used [15]. However, in our offline data analysis, the results of the LASSO-based method were worse than those of the CCA-based method when using eight channels (Figures 2 and 3). We further used the data from O1, O2, and Oz to run the frequency recognition as in the reference [15]. The result was shown in Figure 4. It seems that the LASSO-based method was better than the CCA-based method (consistent with the results in [15]). The proposed LRT-based method was better than the CCA-based method and showed similar performance to the LASSO-based method under most time windows. At this point, we may infer that the LRT-based method can effectively extract robust and detectable features of SSVEP that are interfered by other noises. Although the results were similar for the LRT-based method and LASSO-based method when only using channels O1, O2, and Oz, the former did not require a penalty parameter that is necessary for the latter to generate the desired performance. Accordingly, the LRT-based method is indicated to be a promising candidate for the frequency recognition.

The linear correlation may not extract the nonlinear structure in multichannel EEG signals. To further improve the performance of LRT-based method, we will take into account the nonlinearity between two EEG signals in our future study. In addition, the correlation computed with LRT-based may be used for brain activity analysis in fMRI data [18], EEG data [19], multimodal data [20], and so forth. It may be another important future direction.

The reference signals used in this study were the preconstructed sine-cosine waves according to the stimulus frequencies. These reference signals may fail to provide the subject-specific and intertrial feature information. In order to further improve the accuracy of the frequency recognition methods, two methods, that is, L1-regularized multiway canonical correlation analysis and multiset canonical correlation analysis, were presented to refine the reference signals [21, 22]. For each subject, these two methods generate the optimized reference signals which extract the SSVEP features from the training data. Hence, it is worthy of further study to adopt the refined reference signals in our proposed frequency method to further improve its performance and fuse the existed frequency recognition method to generate more robust and efficient BCI system [13, 15, 23].

The stimulus frequency set may be an important parameter for high performance BCI system. The frequencies may influence the recognition performance to some degree because different subjects may have their favored frequencies [4, 11]. In this study, the 10 Hz was chosen because it is an integer divider of the screen refresh rate (60 Hz) and produces strong SSVEP. In the experiment, we did not observe significant negative effects from the alpha rhythm on SSVEP. One future improvement of the system could be to add a frequency selection procedure to choose the optimal frequency combination for each user. With the frequency selection procedure, we may avoid the possible negative role of the alpha and provide the more efficient BCI system for the users.

In summary, a novel frequency recognition method was proposed based on the LRT, and its efficiency was validated with both simulation data and offline real EEG data. The results indicated that the new method outperformed the popular CCA-based method and the LASSO-based method in some concerned aspects like short time window and robustness to noise. It may be a new promising candidate for frequency recognition to develop SSVEP-based BCI systems with high performance.

## Figures and Tables

**Table 1:** Recognition accuracies (%) for eleven subjects by the three methods with seven different time windows. The better results for each subject are displayed in bold at each time window.

Time	Method	Subjects											Average
		S1	S2	S3	S4	S5	S6	S7	S8	S9	S10	S11	
0.5 s	LRT	**57.1**	**69.6**	**37.9**	**71.7**	**83.3**	90.0	**54.6**	**37.5**	**65.4**	**48.3**	**82.9**	**63.5**
	CCA	42.9	69.2	36.3	64.6	81.3	**90.4**	49.2	33.8	65.0	47.5	79.2	59.9
	LASSO	43.8	46.3	33.8	65.4	60.4	67.5	39.2	31.3	50.8	37.1	42.1	47.0

0.75 s	LRT	**65.6**	**83.8**	**45.6**	**83.1**	**91.9**	**98.1**	**61.9**	**45.0**	**76.9**	**63.1**	**93.8**	**73.5**
	CCA	65.0	81.9	38.1	81.9	88.8	95.6	61.9	40.0	72.5	60.6	91.9	70.7
	LASSO	57.5	61.9	41.3	80.6	82.5	80.0	46.3	30.0	60.6	46.3	53.1	58.2

1 s	LRT	**71.7**	90.8	**50.8**	**88.3**	**95.0**	**98.3**	**74.2**	**51.7**	85.8	**72.5**	**96.7**	**79.6**
	CCA	69.2	92.5	44.2	85.8	90.8	97.5	65.8	47.5	**87.5**	72.5	95.8	77.2
	LASSO	64.2	74.2	44.2	87.5	88.3	89.2	48.3	40.8	65.0	56.7	56.7	65.0

1.25 s	LRT	**82.3**	**93.8**	**57.3**	**91.7**	**99.0**	**100.0**	**80.2**	**53.1**	90.6	69.8	97.9	**83.2**
	CCA	80.2	91.7	47.9	90.6	95.8	99.0	77.1	50.0	**91.7**	**70.8**	**99.0**	81.3
	LASSO	75.0	82.3	43.8	90.6	93.8	91.7	56.3	38.5	75.0	60.4	53.1	69.1

1.50 s	LRT	**88.8**	**96.3**	**61.3**	**93.8**	**100.0**	**100.0**	**82.5**	**58.8**	**95.0**	**76.3**	**98.8**	**86.5**
	CCA	85.0	96.3	53.8	92.5	97.5	100.0	77.5	55.0	93.8	75.0	98.8	84.1
	LASSO	71.3	81.3	56.3	95.0	96.3	95.0	56.3	45.0	75.0	65.0	58.8	72.3

1.75 s	LRT	**88.2**	**98.5**	**60.3**	97.1	**98.5**	**100.0**	85.3	57.4	**97.1**	**89.7**	**100.0**	**88.4**
	CCA	88.2	98.5	54.4	95.6	97.1	100.0	**88.2**	**61.8**	95.6	88.2	100.0	88.0
	LASSO	75.0	86.8	57.4	**100.0 **	98.5	97.1	57.4	51.5	79.4	72.1	55.9	75.5

2 s	LRT	**90.0**	**100.0**	**65.0**	**93.3**	**98.3**	**100.0**	85.0	61.7	**98.3**	85.0	**100.0**	**88.8**
	CCA	88.3	98.3	60.0	93.3	98.3	100.0	**86.7**	**65.0**	98.3	**86.7**	100.0	88.6
	LASSO	83.3	88.3	65.0	98.3	98.3	98.3	70.0	51.7	81.7	76.7	73.3	80.5

**Table 2:** The information transfer rate (bits/min) for 11 subjects by the three methods with seven different time windows. The better results for each subject are displayed in bold at each time window length.

Time	Method	Subjects											Average
		S1	S2	S3	S4	S5	S6	S7	S8	S9	S10	S11	
0.5 s	LRT	**20.1**	**37.9**	**3.5**	**41.5**	**65.1**	**82.4**	**17.2**	**3.3**	**31.3**	**10.9**	**64.1**	**34.3**
	CCA	6.6	37.3	2.7	30.1	60.5	83.5	11.7	1.7	30.7	10.2	56.0	30.1
	LASSO	7.2	9.2	1.7	31.3	24.2	34.5	4.2	0.9	13.2	3.1	6.0	12.3

0.75 s	LRT	**25.3 **	**53.0 **	**6.9 **	**51.7 **	**70.4 **	**88.0 **	**21.0 **	**6.5 **	**41.0 **	**22.3 **	**75.2 **	**41.9 **
	CCA	24.5	49.5	2.9	49.5	63.2	80.2	21.0	3.7	34.3	19.6	70.4	38.1
	LASSO	16.4	21.0	4.4	47.2	50.6	46.1	7.3	0.4	19.6	7.3	12.5	21.2

1 s	LRT	**27.7 **	56.4	**8.8 **	**51.8 **	**65.4 **	**74.0 **	**30.7 **	**9.4 **	47.4	**28.6 **	**69.5 **	**42.7 **
	CCA	24.8	**59.9 **	5.0	47.4	56.4	71.7	21.2	6.8	**50.3 **	28.6	67.3	40.0
	LASSO	19.7	30.7	5.0	50.3	51.8	53.4	7.3	3.5	20.4	13.1	13.1	24.4

1.25 s	LRT	**35.9 **	**53.7 **	**11.6 **	**49.9 **	**65.3 **	**68.6 **	**33.2 **	**8.9 **	48.0	21.9	62.4	**41.8 **
	CCA	33.2	49.9	6.0	48.0	57.7	65.3	29.5	7.1	**49.9 **	**22.8 **	**65.3 **	39.5
	LASSO	27.2	35.9	4.1	48.0	53.7	49.9	10.9	2.2	27.2	13.8	8.9	25.6

1.50 s	LRT	**39.5 **	**51.4 **	**12.7 **	**47.0 **	**60.0 **	**60.0 **	**31.6 **	**11.1 **	**49.0 **	**25.0 **	**56.6 **	**40.4 **
	CCA	34.6	51.4	8.2	44.9	53.8	60.0	26.2	8.8	47.0	23.8	56.6	37.7
	LASSO	20.4	30.3	9.6	49.0	51.4	49.0	9.6	4.1	23.8	15.3	11.1	24.9

1.75 s	LRT	**34.4 **	**49.7 **	**10.7 **	47.1	**49.7 **	**53.3 **	31.1	9.1	**47.1 **	**36.2 **	**53.3 **	**38.3 **
	CCA	34.4	49.7	7.5	44.5	47.1	53.3	**34.4 **	**11.6 **	44.5	34.4	53.3	37.7
	LASSO	21.1	32.7	9.1	**53.3 **	49.7	47.1	9.1	6.2	25.1	18.8	8.3	25.5

2 s	LRT	**32.9 **	**48.0 **	**12.3 **	**36.9 **	**44.4 **	**48.0 **	27.7	10.4	**44.4 **	27.7	**48.0 **	**34.6 **
	CCA	31.1	44.4	9.5	36.9	44.4	48.0	**29.4 **	**12.3 **	44.4	**29.4 **	48.0	34.3
	LASSO	26.0	31.1	12.3	44.4	44.4	44.4	15.4	5.6	24.6	20.3	17.8	26.0
